# The Antiviral Potential of Algal Lectins

**DOI:** 10.3390/md21100515

**Published:** 2023-09-28

**Authors:** Christian Alvarez, Carina Félix, Marco F. L. Lemos

**Affiliations:** MARE-Marine and Environmental Sciences Centre & ARNET—Aquatic Research Infrastructure Network Associated Laboratory, ESTM, Polytechnic of Leiria, 2520-641 Peniche, Portugal; christianandres1@hotmail.com (C.A.); carina.r.felix@ipleiria.pt (C.F.)

**Keywords:** bioactive compounds, lectins, marine biotechnology, macroalgae, mechanisms of action, microalga, seaweed, virus

## Abstract

Algae have emerged as fascinating subjects of study due to their vast potential as sources of valuable metabolites with diverse biotechnological applications, including their use as fertilizers, feed, food, and even pharmaceutical precursors. Among the numerous compounds found in algae, lectins have garnered special attention for their unique structures and carbohydrate specificities, distinguishing them from lectins derived from other sources. Here, a comprehensive overview of the latest scientific and technological advancements in the realm of algal lectins with a particular focus on their antiviral properties is provided. These lectins have displayed remarkable effectiveness against a wide range of viruses, thereby holding great promise for various antiviral applications. It is worth noting that several alga species have already been successfully commercialized for their antiviral potential. However, the discovery of a diverse array of lectins with potent antiviral capabilities suggests that the field holds immense untapped potential for further expansion. In conclusion, algae stand as a valuable and versatile resource, and their lectins offer an exciting avenue for developing novel antiviral agents, which may lead to the development of cutting-edge antiviral therapies.

## 1. Introduction

Viruses are the most abundant biological entities on Earth (though the term “biological” may still offer doubts to some biologists). Their diversity, accompanied by their rapid rates of mutation and adaptability, makes them an important threat to humanity [1]. Although many viruses are considered harmful to humans, only a few can cause serious health problems [2]. To the best of our knowledge, there are more than 1000 known species of viruses that are capable of infecting humans [3]. Antiviral drugs are used for the treatment of viral infections [4]; however, only drugs for ten viral species known to infect humans are clinically approved [5]. This underscores the significance of discovering new sources for antiviral compounds. A diverse range of molecules with anticancer, antiviral, and antibiotic activities has been identified and isolated from algal species. In fact, algae represent one of the richest known sources of natural antivirals, as well as a significant source of other bioactive compounds [6].

## 2. Algal Antiviral Compounds

Algae comprise two large groups: macroalgae and microalgae. Both are widely explored photosynthetic organism groups since they contain a plethora of bioactive compounds in their composition [7]. Currently, algae have become organisms of special attention since they are capable of growing in almost every habitat on Earth, show an extraordinary biological diversity with more than 200,000 described species [8], and can be used as fertilizers, foods, and sources of active metabolites, among others [9]. They have also emerged as a next-generation resource with the potential to address the urgent demands of the growing pharmaceutical, agricultural, and other industries [8].

A comprehensive body of research on algal compounds has developed over the years, with the earliest reference dating back to 1958. This field has seen a consistent increase in both publications and patents worldwide, a trend that was extensively reviewed by Pagarete and colleagues for the period up to December 2020 [10].

Numerous algal metabolites exhibiting antiviral activity against a wide spectrum of viruses belonging to 28 different viral families, totaling 61 distinct viruses, have been documented. Nevertheless, it is worth noting that the bulk of published research pertains to retroviruses and herpesviruses, a trend that was also elucidated by Pagarete et al. in their comprehensive review [10].

Liu et al. [6] conducted a comprehensive review highlighting the active antiviral properties exhibited by various metabolites derived from different algae. Brown seaweeds belonging to the *Sargassum* genus have been historically employed in the treatment of fever and other infections caused by viruses [11]. From red algae, carrageenan, galactan, and other extracts have proven potential antiviral capacities against pathogenic viruses, including human papillomavirus (HPV), human immunodeficiency virus (HIV), hepatitis A virus (HAV), human rhinovirus (HRV), alfalfa mosaic virus (AMV), and influenza viruses [12]. Alginate and some of its derivates have demonstrated activity against HIV and hepatitis B virus (HBV) [13]. From diatomea, a compound known as naviculan has potential activity against herpes simplex virus (HSV) [14]. From dinoflagellates, polysaccharides have potential inhibitory effects against encephalomyocarditis virus (EMCV), respiratory syncytial virus (RSV), parainfluenza, and influenza A and B viruses [15]. From *Nostoc*, the compound nostaflan inhibits influenza A virus, HSV_1_, HSV_2_, and human cytomegalovirus [16]. It is important to highlight that, despite the substantial in vitro potential of polysaccharides as antiviral agents, no successful clinical trials have been realized. In fact, protocols for removing polysaccharides from natural aqueous extracts have been utilized due to their nonspecific binding to proteins, in order to isolate the most promising compounds for combating viruses such as HIV [17].

## 3. Lectins

Lectins, a class of molecules prevalent in various algae, are renowned for their antiviral properties and offering promising avenues for the development of novel pharmaceuticals [10]. In the current study, we delve into the primary groups extracted from algae that hold potential as antiviral agents (Table 1).

These are natural, bioactive proteins of non-immune origin, characterized by their remarkable capacity to bind polysaccharides, glycans, glycoproteins, and glycolipids with a high degree of specificity [18,19]. These compounds hold significant importance owing to their biological properties, which include interactions with specific blood groups such as lymphocyte agglutination, sperm, erythrocytes, and platelets. Additionally, they can induce mitosis in lymphocytes and exhibit the capability to induce cytotoxic effects on T-lymphocytes [20]. They possess two or more carbohydrate-binding sites that have the ability to agglutinate erythrocytes without altering the properties of carbohydrates [21,22]. Some applications include the assessment of lymphoproliferative and cytotoxic functions in mononuclear cells, the detection of chromosomal abnormalities, the utilization of fluorescent markers to investigate structural alterations in glyco-conjugates located on cell surfaces, and other applications in antiviral research [22]. Algal lectins are presently employed in biomedical research for a range of purposes, including their roles in antiviral, antinociceptive, anti-inflammatory, and antitumor activities, among other applications [19,21].

The origins of lectin research trace back to 1888, when the agglutination of red blood cells by ricin was first demonstrated. This compound found notoriety during the World Wars as a weapon of choice [23]. The term lectin was introduced by Boyd in 1954 and derives from the Latin word “legere”, which means “to choose” [24]. The first pure hemagglutinin was obtained from a type of bean in 1919 and is the most studied lectin to date [20,24]. The initial documentation of lectins being employed as antiviral agents dates back to the 1980s, a crucial period during the emergence of HIV. During this time, research showcased their capacity to inhibit reverse transcriptase in HIV-1 patients, positioning lectins as significant contenders in the realm of antiviral treatments [24].

Regarding innate immunity, lectins can function as Pattern Recognition Receptors (PRRs), enabling them to identify Pathogen-Associated Molecular Patterns (PAMPs) and Damage-Associated Molecular Patterns (DAMPs). The principal classes of lectins involved in innate immunity include C-type lectin receptors (CLRs), siglecs, and galectins [25]. For instance, galectins (carbohydrate (glycan)-binding proteins that are expressed by a wide range of cells and bind to galactose-containing glycans) can modulate not only innate but also adaptive immune cells through binding to glycans on the surface of immune cells or intracellularly (by carbohydrate-dependent or -independent interactions). This family of lectins expressed by immune cells can also be involved in host responses to infection. They have the capacity to directly bind to microorganisms and can modulate various antimicrobial functions, including processes like autophagy [26].

These different classes of lectins exhibit diverse functions in antimicrobial defense and immune homeostasis, making the use of lectins a promising strategy for modeling immune responses in contexts such as infections, autoimmunity, cancer, and vaccination [25].

### 3.1. Algal Lectins

Algae represent an extensive reservoir of lectins characterized by their distinctive properties [27]. Certain alga species have been documented to harbor lectins that exhibit carbohydrate specificity towards complex glycoproteins or high-mannose N-glycans [27]. These interactions give them the ability to bind terminal mannoses present in high-mannose oligosaccharides and crosslink these glycans on the surface of the viral envelope glycoprotein gp120 [28]. The presence of lectins has been demonstrated in about 838 alga species, according to Web of Science. However, it is anticipated that this number will continue to grow, given the existence of thousands of marine alga species worldwide yet to be studied.

Lectins are classified according to their structure into the following: merolectins, possessing one carbohydrate recognition domain (CRD), small proteins that due to their monovalent nature are unable to precipitate glycoconjugates or agglutinate cells; hololectins, containing two or more CRDs with homologous structure; chimeric lectins, fusion proteins that have a CRD in conjunction with a domain with catalytic activity; and super lectins, possessing at least two different CRDs [23].

#### Production of Algal Lectins

Some algae can be cultivated in photobioreactors, such as bubble column, airlift, stirred tank, and tubular recycle photobioreactors [29]. The most important prerequisites for mass-producing lectins are the identification of sources with elevated lectin content and the implementation of straightforward purification methods [30]. The presence of cell walls and intracellular polysaccharides causes high viscosity and ionic interactions, making the extraction process difficult, as does some features of the morphology of the marine algae themselves; for example, marine algae with a tougher thallus may require more processing [31]. Lectins are typically isolated from marine algae by grinding the algal tissue with liquid nitrogen and extracting with appropriate buffers and alcohol, as described by Maliki and co-workers [32]. This method can produce higher yields, but it is not ideal for large-scale production, since a large amount of biomass is required to extract a small quantity of compound and a significant amount of waste is generated during the extraction process [32,33]. The products of biosynthesis also depend on several other factors such as compound sensitivity to shear forces, the tissue growth rate, cell aggregation, and cell flotation [32]. As an alternative to invasive and destructive techniques, the cold steeping infusion (CSI) method is available. This allows the recovery of lectins from algal cultures without causing harm to the producing tissues. In this case, algae release extracellular bioactive compounds—lectins included—that are continuously retrieved from buffer solutions, allowing the continuous growth of the algal biomass to subsequent culture cycles [34].

### 3.2. Algal Lectins with Antiviral Potential

#### 3.2.1. Griffithsin 

The lectin griffithsin (GRFT) is a red-alga-derived lectin known as a potent antiviral agent capable of preventing and treating infections caused by several enveloped viruses. GRFT is thermostable and resists a large pH range, exhibits little toxicity or immunogenicity, and is available for large-scale manufacturing [35].

**Figure 1:**
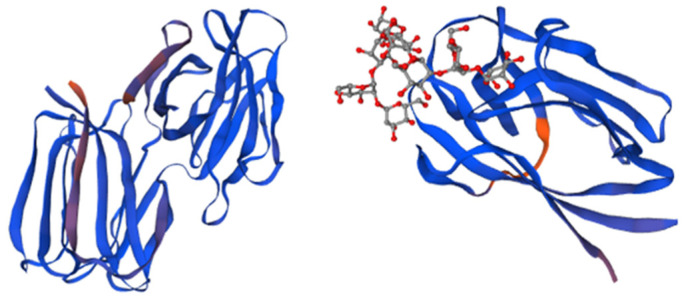
The structure of Griffithsin and the domains of this protein. Amino acids located within beta strands are highlighted in magenta to indicate their secondary structure, while each monomer of the domain-swapped dimer is depicted in blue. The crystal structure of a GRFT dimer with six mannoses is depicted, with each GRFT monomer shown in blue, and the N-terminal extension resulting from the cloning procedure colored in orange. Adapted from Pubmed and Micewicz et al. [41] (PDB entry code 2GTY).

Griffithsin was isolated for the first time from an aqueous extract of *Griffithsia* sp. (Rhodophyta). It is a protein widely known for its ability to inhibit HVI_1_ in vitro. This compound is considered one of the most powerful entry inhibitors to date [10]. Its antiviral activity is correlated to its structural characteristics since it presents a unique structure (Figure 1) [28] that is extremely selective to a sugar present in the envelope of many pathogenic viruses, such as hepatitis C virus (HCV), herpes simplex virus 2 (HSV_2_), Japanese encephalitis virus (JEV), and porcine epidemic diarrhea virus (PEDV) [36]. Griffithsin is not only an extremely effective HIV entry inhibitor, but also improves antibody responses and avoids cell fusion and cell-to-cell transmission of HIV [37]. According to an in silico study by Naik and colleagues [38], lectins have a high binding affinity for the glycans of the SARS-CoV-2 spike glycoprotein (found on the surface of some enveloped viruses). These authors found that an interaction between the model lectin Lablab purpureus and the amino acid residues Asn487, Tyr489, Gln493, Lys417, and Tyr505 of the receptor binding domain (RBD) of SARS-CoV-2 was formed, and an analogous interaction for SARS-CoV-2 spike protein was observed with griffithsin, demonstrating the potential of these molecules for neutralizing coronavirus infection [38].

Although lectins are known for their broad-spectrum activity, high specificity, and local delivery (topical application), there are also concerns regarding their toxicity, as they can induce mitogenic activity after long exposures. Among the broad range of assessed lectins, griffithsin (GRFT) distinguishes itself in several aspects. Notably, it deviates from the norm with its lack of mitogenic activity. Furthermore, it has demonstrated promising outcomes in Phase 1 clinical trials, confirming its safety when used in topical formulations. Additionally, it holds the potential for cost-effective production in substantial quantities [39,40].

Griffithsin’s antiviral activity stems from its ability to bind terminal mannoses present in high-mannose oligosaccharides and crosslink these glycans on the surface of the viral envelope glycoproteins [28]. GRFT is capable of inhibiting gp120 (a glycoprotein that is part of the outer layer of the virus) from binding to the 2G12 mAb, which targets N-linked glycans at positions 332, 339, and 392 on gp120.

The mechanisms of action are based on the exposure of the CD4 binding site of gp120 through the glycan at position 386 and blockage of the coreceptor binding step [37]; inhibition of mannose binding to gp120 and improvement of the humoral immune response to gp120 [42]; inhibition of gp120 binding to DC-SIGN and expulsion of gp120 from the gp120/DC-SIGN complex [43]; alteration of the gp120 structure through the exposure of the CD4 binding site [43]; intra-virion crosslinking of gp120 [35]; and inter-virion aggregation or clustering of gp120 [29].

Using the GRFT molecule as a base, Micewicz et al. [41] successfully constructed a model for a peptide referred to as grifonin1. This peptide consists of three covalently linked beta sheets, which exhibit a distinctive triple symmetry. Even though it shows a less potent antiviral activity than its base protein, it is capable of inhibiting viral cycles at low concentrations. To enrich the capacity of GRFT to inhibit HIV infection, Kagiampakis et al. [44] created a covalently linked fusion protein based on GRFT and a notorious virus entry inhibitor known as GRFT-C37. GRFT-C37 is capable of blocking virus fusion by binding to the terminal helices of the gp41, with a capacity between 5 and 8 times greater than that of GRFT [43,45].

#### 3.2.2. Cyanovirin-N

Cyanovirin-N (CV-N) is a 101-amino-acid protein extremely resistant to physicochemical degradation, and it is capable of supporting treatment with denaturants, detergents, organic solvents, and heat up to 100 °C with no apparent loss of antiviral properties [46].

**Figure 2:**
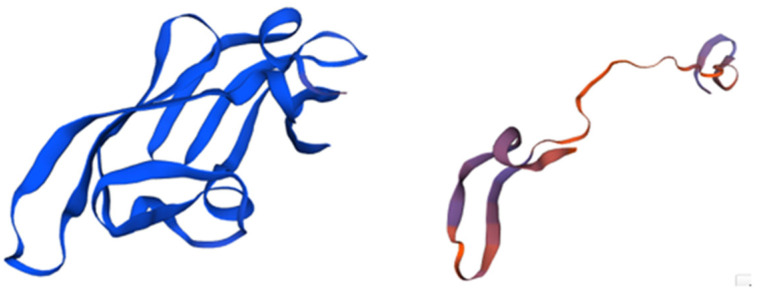
The structure of Cyanovirin and its domains. The protein, consisting of 101 amino acids, is depicted in blue, with its β-strands and helical turns also highlighted in blue. The stereo view shows superpositions of the ensemble of the final 40 simulated annealing structures of cyanovirin-N. The backbone is shown in magenta, the disulfide bridges are depicted in orange, and all other side chains are represented in blue. Adapted from Pubmed and Botos et al. [50] (PDB entry code 1M5J).

This compound was initially isolated in 1997 from the cyanobacterium *Nostoc ellipsosporum* (Cyanobacteria) as part of efforts to identify agents from natural sources capable of inhibiting human immunodeficiency virus (HIV) infection [47,48]. The antiviral activity of CV-N is closely linked to its structural characteristics. Specifically, it possesses a duplicated internal sequence that includes four cysteine (Cys) group residues, leading to the formation of two disulfide bridges. This structural arrangement is depicted in Figure 2 [49]. These bonds are fundamental for the stabilization of the protein’s structure, determining its antiviral activity.

Cyanovirin has demonstrated antiviral activity against several viruses, including HIV-1 and HIV-2. It achieves this by inhibiting cell fusion and interrupting the viral transmission cycle through its binding to gp120, a glycoprotein that constitutes a part of the outer layer of the virus and is found in the viral envelope **[51]**.

CV-N interacts with high-mannose oligosaccharide structures on gp120 and gp41 and is thus capable of inactivating various virus strains. CV-N possesses two carbohydrate-binding sites, namely, 186 and 189, showing the highest affinity for oligomannose oligosaccharides such as Man8 and Man9. Several studies revealed that the binding of a nonamannoside to CV-N was multivalent in nature, and nonamannoside was found to cross-link CV-N molecules through this multivalent binding [52]. CV-N binds to gp120 in a manner that does not occlude the CD4 binding site or other domains on gp120 and does not interfere with soluble CD4-induced conformational changes in gp120. CV-N could prevent essential interactions between the envelope glycoprotein and target cell receptors acting at the level of the virus and not the target cell, to abort the initial infection process [53]. CV-N shows enhanced cytotoxicity effects against HIV-infected gp120-expressing H9 cells and exerts its activity by binding to high-mannose oligosaccharides, located predominantly in the C2–C4 region of gp120, preventing the virus from entering by blocking the fusion with the cell membrane and cell-to-cell transmission [54].

Using the cyanovirin molecule as a base, Lei et al. [55] modeled a peptide by modifying the α-amine group of the N-terminus of LCV-N (linker cyanovirin) with 10 kDa polyethylene glycol propionaldehyde (mPEG-ALD). The new compound, known as a monoPEGylated derivative of CV-N, exhibits potent inhibitory activity against acyclovir-resistant strains of the herpes simplex virus (HSV), with IC_50_ values in the nanomolar (nM) range.

#### 3.2.3. Scytovirin 

**Figure 3:**
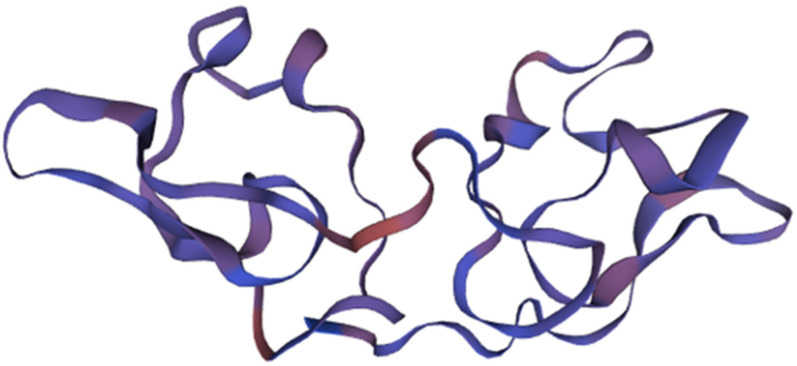
The structure and domains of scytovirin. Structural domain 1 is represented in blue, structural domain 2 is represented in purple, and the disulfide bonds are highlighted in magenta. Adapted from Pubmed and Moulaei et al. [59] (PDB entry code 2JMVJ).

Scytovirin (SVN) is a cyanobacterium-derived carbohydrate-binding protein formed by a single chain of 95 amino acids. It has demonstrated very low toxicity in both human hepatocyte carcinoma cell lines and mouse models [56]. This compound was isolated for the first time in 2007 from *Scytonema varium* (Cyanobacteria), strain HG-24-1, by McFeeters et al. [57]. Its antiviral activity is correlated to the two sequence repeats, forming two identical structural domains, SD1: 3-43 and SD2: 51-89, divided by a pro-rich linker [58]. Each domain contains three aromatic amino acids involved in carbohydrate binding and two intra-domain disulphide bonds. The fifth inter-domain disulphide bond links Cys-7 and Cys-55. The two carbohydrate-binding domains are specific for Man α (1-2)Man α (1-6)Man α (1-6)Man tetramannose, as shown in Figure 3, conferring the ability to block viral and fungal glycans with high mannose content [10,59].

SVN has two carbohydrate-binding sites with substantially different affinities, capable of binding to the envelope glycoproteins gp160, gp120, and gp41, but it does not bind to the T-cell extracellular CD4 receptor or to other common cell surface proteins [57].

#### 3.2.4. Microvirin

**Figure 4:**
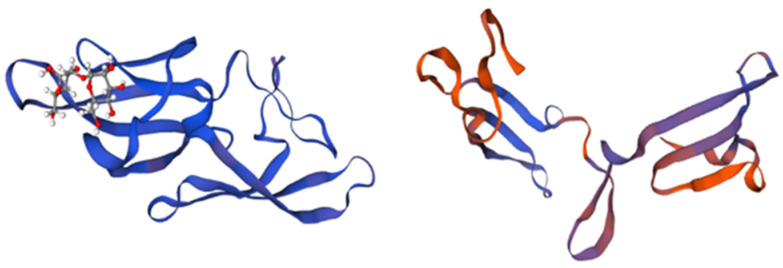
Structure and domains of microvirin. The structure is divided into two structural domains, depicted in blue and magenta, while the bound glycan is colored in orange. The insertion of four amino acids in domain A, as compared to domain B, is indicated in blue and magenta. Adapted from Pubmed and Shahzad-ul-Hussan et al. [61] (PDB entry code 2y1s).

Microvirin (MNV) is an α (1,2)-mannose-specific lectin that possesses anti-HIV activity comparable to that of cyanovirin. However, it has been shown to be 50 times less cytotoxic [60]. This 108-amino-acid-long lectin is a monomer in solution with a single glycan-binding site that also recognizes terminal α (1,2)-mannose sugars, as presented in Figure 4 [61].

Microvirin was first extracted from *Microcystis aeruginosa* (Cyanobacteria), a species known for forming algal blooms [60], and it displayed a robust affinity with certain viruses [62]. It also inhibits syncytium formation (the accumulation of infected cells with neighboring cells, leading to the formation of multinucleate enlarged cells) between persistently HIV-1-infected T cells and uninfected CD4(+) T cells and inhibits transmission to CD4(+) T cells [61]. It irreversibly inactivates a wide range of immunodeficiency viruses, such as HIV-1 and HIV-2 [51].

Using the MVN molecule as a base, Shahid et al. [62] modeled a variant with two domains exhibiting 100% sequence identity. This alteration served to decrease the chemical heterogeneity of the molecule. The new compound, known as LUMS1, has potent inhibitory activity against HIV-1 and HCV, showing EC_50_ values of 37.2 and 45.3 nM, respectively [62]. There is also an inhibitory role of MVN during the attachment of gp120 to cellular receptors and subsequent fusion steps. This compound has preference for the co-receptor CCR5, the main co-receptor used by HIV for transmission [63,64].

#### 3.2.5. Others

##### The Brown-Alga-Derived OAAH (*Oscillatoria agardhii* Agglutinin Homolog) Lectin Family

The *Planktothrix agardhii* (formerly *Oscillatoria agardhii*) (Cyanobacteria) agglutinin homolog (OAAH) is a family of proteins belonging to lectins with a sequence repeat of 66 amino acids [65]. The first member of this family was isolated for the first time in 2011 from *Planktothrix agardhii,* by Koharudin et al. [64]. These lectins can withstand a wide pH range from 4 to 11 and high temperatures, remaining active even at temperatures of at least 80 °C [66]. They are recognized for their capacity to inhibit HIV [27].

The antiviral activity of these lectins is closely tied to their structural characteristics. Specifically, they feature 10 β-strands that fold into a single, compact, β-barrel-like domain, resulting in a distinctive topology characterized by two symmetric carbohydrate-binding sites with a preference for man α(1–6)man-linked sugars [65]. The OAAH family proteins also interact with high-mannose oligosaccharide structures on gp120; thus, they are capable of inactivating several virus strains [27].

##### The Yellow-Alga-Derived Legume-Lectin-like Family

This lectin family possesses properties that are effective against HIV, and it also shows potential utility in anticancer applications [27]. The scaffold found in legume lectins (Fabaceae) also occurs in various alga species, including *Ostreococcus tauri* (Chlorophyta), *Gracilaria fisheri* (formerly *Hydropuntia fisheri*) (Rhodophyta), *Microchloropsis gaditana* (formerly *Nannochloropsis gaditana*) (Eustigmatophyceae), and *Porphyra umbilicalis* (Rhodophyta) [27]. They have two undistinguishable mannose-binding sites and differ from the single-chain legume lectins, which results from the non-covalent association of four protomers to give homotetrameric mannose-binding lectins [67]. Their antiviral activity is correlated to their affinity for mannose and high-mannose glycans forming complexes with glycoproteins or high-mannose N-glycan [27]. These interactions give them the ability to bind the terminal mannoses present in high-mannose oligosaccharides and crosslink these glycans on the surface of the viral envelope glycoproteins [28].

##### Green-Alga-Derived *Galanthus nivalis* Agglutinin (GNA)

Monocot-lectin or GNA-like are a family of lectins containing three bundles of four β-strands arranged into a flattened β-prism structure around a central pseudoaxis. The GNA-like seaweed lectin from the brown alga *Boodlea coacta* showed inhibitory activity against HIV-1 and influenza virus H_1_N_1_ [27]. Their antiviral activity is correlated to their carbohydrate-binding propensity, conferring the ability to block viral and fungal glycans with high mannose content. Their high affinity for HIV contributes to the potency of their antiviral activity, presenting an EC_50_ of 8.2 nM, improving the antibody responses and avoiding cell fusion and HIV cell-to-cell transmission [68].

##### The Multifunctional Protein in Peroxisomal β-Oxidation (MFP2)-like Families

The lectin from the green alga *Bryopsis plumosa* has been characterized as a Man-specific lectin structurally related to the Multifunctional Protein in Peroxisomal β-Oxidation (MFP2), consisting of three β-harpins that adopt a triangular disposition to form a sort of β-trefoil structure, presenting antiviral activity against HIV [27]. Their antiviral activity is correlated to the presence of a network of hydrogen bonds of mannose and amino acids Lys123, Asp125, Ser154, Asp163, and Val164, forming a monosaccharide-binding pocket [27]. This interaction improves the antibody responses and avoids cell fusion and cell-to-cell transmission of HIV [27].

##### Mannose-Binding Lectin from *Grateloupia chiangii* (*G. chiangii* Lectin, GCL)

In 2020, Hwang et al. [69] purified a novel mannose-binding lectin from the macroalga *Grateloupia chiangii* (Rhodophyta) (GCL) using antiviral screens and affinity chromatography. Its activity could be assumed due to its specificity for high-mannan N-glycans, which are related to antiviral abilities. This interaction can confer the ability to block viral and fungal glycans with high mannose content. The preliminary tests demonstrated antiviral activity of GCL against the influenza virus and HSV but not against HIV [69]. However, further investigation is necessary to fully understand the behavior of this lectin.

**Table 1:** Summary of lectins extracted from algae with antiviral potential, outlining their specificity and the corresponding targets they interact with.

Alga	Lectin	Specificity	Virus	Reference
*Griffithsia* sp. (Rhodophyta)	Griffithsin	Mannose	HIV	[37,70]
			HSV	[36]
			HCV	[29]
			SARS-CoV_1_ and MERS	[71,72]
			EBOV	[73]
			JEV	[37]
			HPV	[36]
*Nostoc ellipsosporum* (Cyanobacteria)	CV-N	High-mannose glycans	HIV	[74]
			HCV	[75]
			Influenza virus	[76]
			Rhinoviruses	[59]
			SARS-CoV_2_	[77]
			EBOV	[52]
			Measles virus	[55]
			HHV_6_	
			SIV	[52]
			*Trichomonas vaginalis*	[78]
*Cytonema varium* (Cyanobacteria)	SVN	High-mannose glycans	HIV	[79]
			HCV	
			SARS-CoV_1_	[77]
			EBOV	[79]
*Microcystis viridis* and *Microcystis aeruginosa* (Cyanobacteria)	MVN	High-mannose glycans	HIV-1	[62]
			HCV	
*Oscillatoria agardhii* (Cyanobacteria)	OAAH	High-mannose glycans	HIV-1	[80]
*Ostreococcus tauri* (Chlorophyta), *Gracilaria fisheri* (Rhodophyta), *Microchloropsis gaditana* (Eustigmatophycae), and *Porphyra umbilicalis* (Rhodophyta)	Yellow-alga-derived legume-lectin-like family	Mannose and high-mannose glycans	HIV	[28]
*Boodlea coacta* (Chlorophyta)	Green-alga-derived Galanthus nivalis agglutinin (GNA)	High mannose (HM)-type N-glycans	HIV	[28]
			H_1_N_1_	
*Bryopsis plumosa* (Chlorophyta)	MFP2-like families	Mannose and high-mannose glycans	HIV-1	
*Grateloupia chiangii* (Rhodophyta)	Mannose-binding lectin from *Grateloupia chiangii* (*G. chiangii* lectin, *GCL*)	High-mannan N-glycans	HSV	[69]

## 4. Materials and Methods

This literature review includes the available information regarding lectins from algae with antiviral capacities up to the 13th of July of 2023, using the SCOPUS, WEB OF SCIENCE, and NCBI databases. The search was performed using the keyword combination “Antiviral* AND (Lectins* OR Algae* AND (Macroalga* OR seaweed)” to compile the works including alga extracts with antiviral potential/activity against viruses. In addition, for the review of works reporting the lectin production of algae, the keyword combination “Algae* OR macroalgae* OR microalgae* OR seaweed AND (Lectin OR production* OR * OR content*) AND (virus*) AND (Macroalga* OR seaweed)” was used. For protein visualization and design, sequences were downloaded from NCBI Structure and were designed and visualized using Biozentrum’s SWISS-MODEL online database.

## 5. Conclusions

The study of antiviral activities within algae has encompassed a broad range of viral diseases. However, the major focus of research investment has been directed towards HIV, a virus that has afflicted millions of people worldwide, with an estimated 37 million people carrying the virus. As one of the most extensively studied viruses globally, HIV has prompted significant efforts to explore potential antiviral agents from diverse sources, including algae. Notably, almost all classes of algae have shown remarkable antiviral potential, making them distinctive resources for the detection and development of new antiviral drugs targeting a wide range of viruses. Lectin proteins have emerged as pivotal contributors in this undertaking, showcasing remarkable antiviral capabilities. Two prominent examples are cyanovirin and griffithsin, which have emerged as main antiviral references and hold immense promise as potential treatments against viruses like HIV. Among the different groups of seaweeds, the Rhodophyta and Phaeophyta groups have been more extensively scrutinized in comparison to Chlorophyta. The substantial number of patents registered in this domain underscores the keen interest of major economic powers in harnessing the potential of alga-based antiviral solutions. As research continues to progress, the field of alga-based antiviral agents is constantly expanding, with a growing number of new research initiatives and patents. Encouraging clinical trials are already in progress, offering the potential for the development of alga-based treatments for challenging viruses, such as HIV and coronaviruses. While none of these compounds have made it to the market thus far, the evident interest in lectins underscores their pivotal role as potential drug candidates. The ongoing exploration of algae and their antiviral potential holds significant promise for unveiling novel therapeutic options to combat viral infections. This provides hope for the enhancement of healthcare outcomes in the future.
